# A Role for Nonsense-Mediated mRNA Decay in Plants: Pathogen Responses Are Induced in *Arabidopsis thaliana* NMD Mutants

**DOI:** 10.1371/journal.pone.0031917

**Published:** 2012-02-22

**Authors:** Samantha Rayson, Luis Arciga-Reyes, Lucie Wootton, Marta De Torres Zabala, William Truman, Neil Graham, Murray Grant, Brendan Davies

**Affiliations:** 1 Centre for Plant Sciences, Faculty of Biological Sciences, University of Leeds, Leeds, United Kingdom; 2 School of Biosciences, University of Exeter, Exeter, United Kingdom; 3 Nottingham Arabidopsis Stock Centre, School of Biosciences, University of Nottingham, Sutton Bonington Campus, Loughborough, United Kingdom; University of Melbourne, Australia

## Abstract

Nonsense-mediated mRNA decay (NMD) is a conserved mechanism that targets aberrant mRNAs for destruction. NMD has also been found to regulate the expression of large numbers of genes in diverse organisms, although the biological role for this is unclear and few evolutionarily conserved targets have been identified. Expression analyses of three *Arabidopsis thaliana* lines deficient in NMD reveal that the vast majority of NMD-targeted transcripts are associated with response to pathogens. Congruently, NMD mutants, in which these transcripts are elevated, confer partial resistance to *Pseudomonas syringae*. These findings suggest a biological rationale for the regulation of gene expression by NMD in plants and suggest that manipulation of NMD could offer a new approach for crop protection. Amongst the few non-pathogen responsive NMD-targeted genes, one potential NMD targeted signal, the evolutionarily conserved upstream open reading frame (CuORF), was found to be hugely over-represented, raising the possibility that this feature could be used to target specific physiological mRNAs for control by NMD.

## Introduction

Nonsense-mediated mRNA-decay (NMD) is a conserved eukaryotic system that performs two major functions. It identifies aberrant mRNA molecules carrying premature termination codons and targets them for destruction, thus preventing the accumulation of potentially harmful truncated proteins [1]. However, NMD also regulates the expression of normal transcripts; microarray analyses of cells and organisms depleted in various NMD effectors have shown that the expression levels of 1–10% of all genes are affected by NMD in yeast, flies, mammals and plants [2]–[6].

The mRNA features that enable the NMD machinery to identify a termination codon as premature are not yet fully defined and seem to vary between organisms. In mammals, Zebrafish (*Danio rerio*) and *Arabidopsis*, the presence of an exon junction complex (EJC) on the mRNA, downstream of a stop codon, can make it a substrate of NMD [7]–[9]. In *Saccharomyces cerevisiae*, Hrp1p bound to a downstream sequence element can lead to NMD of the *PGK1* transcript [10], [11]. In all organisms, a large distance between a termination codon and the poly(A) tail can also target an mRNA to NMD [9], [12]–[16]. These different mRNA features are targeted by NMD, via a complex containing NMD effector proteins.

Several effectors of NMD have been identified, including the UPFRAMESHIFT1, 2 and 3 and SMG1, 5, 6 and 7 proteins. Mammalian NMD is dependent upon the cyclic phosphorylation and dephosphorylation of UPF1 [17]. The formation of a complex including UPF1, 2 and 3, bound to target mRNA, enables the phosphorylation of UPF1 by SMG1 [17]–[20]. The subsequent dephosphorylation of UPF1 is mediated by either SMG5/7 or SMG 6 [21]–[24]. The UPF proteins are well conserved [25] and UPF1 is indispensable for NMD in all eukaryotes studied [26], [27]. In contrast, the SMG1 and SMG5-7 proteins are less well conserved. Some organisms that have a functional NMD pathway lack an SMG1 orthologue and/or homologues of one or two of the SMG5-7 proteins [28]. *Arabidopsis* homologues of *UPF1* [29], [30], *UPF2* [9], *UPF3* [31] and *SMG7* [32] have been identified as NMD effectors, but there are no obvious *Arabidopsis SMG1*, *5* or *6* homologues.

Despite the finding that NMD regulates the levels of multiple transcripts in every system studied to date, it has been problematic to identify a biological role for such a mechanism of gene regulation. In mammals, rates of NMD can vary in a tissue-specific manner [33] and in response to hypoxia [34]. Other findings suggest an involvement of NMD in the regulation of responses to exogenous change. For example, the elevation of specific transcripts in response to reactive oxygen species in *Saccharomyces pombe* is dependent on a functional UPF1 [35] and amino acid starvation in mammalian and *Drosophila melanogaster* cells leads to the up-regulation of several NMD target mRNAs [3]. More recently, it has been shown that NMD can be specifically suppressed in differentiating neuronal cells by the expression of a microRNA that targets *UPF1* and a component of the EJC [36]. This local suppression of NMD results in a widespread change in the gene expression profile and is compatible with a role for NMD in normal brain development.

In most model systems it is still unclear why so many genes are subject to post-transcriptional control by NMD. In plants, there are some specific examples of NMD gene regulation either by regulation of alternative splicing in favor of transcripts harboring NMD target features [37]–[39], or by spatial regulation of factors essential for NMD of a sub-set of *Arabidopsis* targets [40]. Taken together, these findings indicate a potential for additional roles of plant NMD in the dynamic regulation of gene expression. There is, however, currently no known biological rationale for the NMD regulation of non-aberrant transcripts in plants. *Arabidopsis* mutants, impaired in NMD, are available for *UPF1*, *UPF3* and *SMG7* [29], [32]. To identify the physiological processes regulated by NMD in *Arabidopsis*, we determined the common transcripts that are enriched in *upf1-5*, *upf3-1* and *smg7-1* mutant plants. Strikingly, the vast majority of such transcripts are associated with pathogen response.

Plants represent a source of nutrients for the microorganisms to which they are exposed. Most potential invaders are kept at bay by innate defences, including both physical and chemical barriers, and inducible defences that respond to pathogen (or microbe) associated molecular patterns (PAMPs/MAMPs), such as flagellin or lipopolysaccharide [41], [42]. PAMP-triggered immunity (PTI) in response to bacterial infection involves rapid host transcriptional reprogramming, callose deposition and production of reactive oxygen species [43]–[46]. Some pathogens have evolved avirulence factors, which confer the ability to evade host pattern recognition receptors or to otherwise suppress PTI [47]. In some cases, host plants have evolved corresponding genes, which allow them to respond to specific avirulence factors by launching the hypersensitive response, in which infected cells and tissues are sacrificed in order to restrict growth and spread of the pathogen [41]. The hypersensitive response commonly precedes systemic acquired resistance (SAR), whereby distal tissues are primed and respond more readily to pathogens. Salicylic acid (SA) is involved with resistance to biotrophic and hemi-biotrophic pathogens and the establishment of SAR [48], [49]. Jasmonic acid (JA) and ethylene are associated with resistance to necrotrophic pathogens [48]. ABA, which has a role in plant responses to several abiotic stresses, is a negative regulator of defense responses [50]. Here, we show that *Arabidopsis* NMD mutants constitutively express a set of pathogen response genes, overproduce salicylic acid and consequently show partial resistance to *Pseudomonas syringae* pathovar tomato DC3000 (DC3000). This indicates a role for NMD in the prevention of inappropriate expression of this suite of genes. Furthermore, we identify a smaller subset of NMD targets that are not induced by pathogen infection but which show an overrepresentation of evolutionarily conserved upstream open reading frames. These findings suggest a biological role for NMD in pathogen response and provide evidence for differential regulation of sets of NMD targets in plants.

## Results

### NMD regulates the abundance of hundreds of *Arabidopsis* transcripts

**Figure 1:**
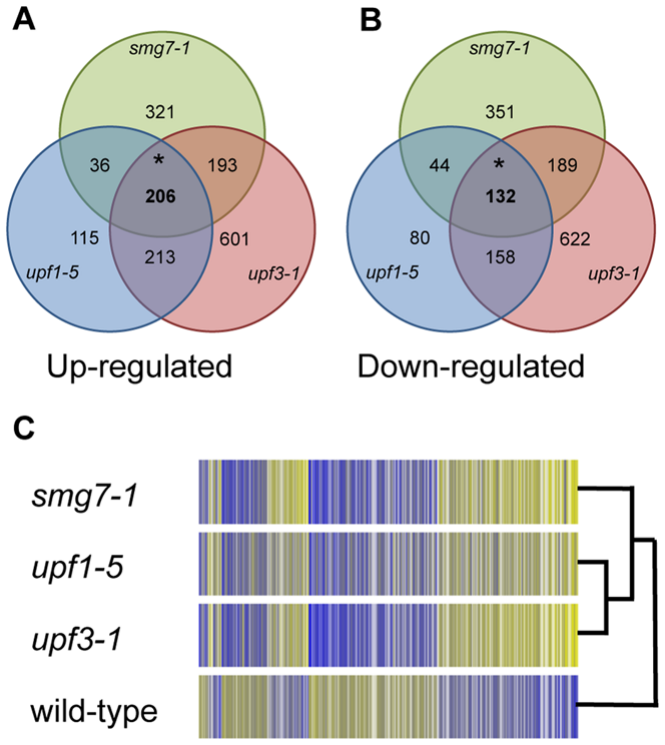
Gene expression in wild-type and NMD mutant *Arabidopsis* plants. (A and B) Genes that are up-regulated and down-regulated respectively in NMD mutant plants when compared to wild-type (p<0.05, fold change >1.5). * The overlap of up or down-regulated genes is greater than would be predicted by chance (p<0.05). (C) Condition tree of all genes that are differentially regulated in at least one of the NMD mutants when compared to wild-type (p<0.05, fold change >1.5). Strongly expressed genes are coloured bright yellow and weakly expressed genes are coloured dark blue.

To determine the effects of NMD on gene expression, microarrays were used to profile transcript abundance in soil-grown *Arabidopsis* wild-type, *upf1-5*, *upf3-1* and *smg7-1* mutant seedlings. For each mutant line, a volcano plot was used to identify transcripts that differed in expression from wild-type significantly (p≤0.05) and by at least 1.5-fold. In the *upf1-5*, *upf3-1* and *smg7-1* mutants respectively, 570, 1213 and 756 transcripts are up-regulated (2.5–5.3% of transcripts represented on the array) (Figure 1A) and 414, 1,101 and 716 are down-regulated (1.8–4.8%) (Figure 1B). A condition tree was built using the expression data for these genes (Figure 1C). The *upf1-5* and *upf3-1* expression profiles show no significant differences from each other. The expression profile of *smg7-1*, although more similar to the profiles of the other NMD mutants than to wild-type, shows a greater degree of difference (p≤0.05), suggesting that SMG7 has additional function(s) distinct from its role in NMD. The genes that are up or down regulated in each mutant were then compared (Figure 1A and B). 206 and 132 of the transcripts (0.9 and 0.6% of transcripts represented on the array) are commonly up or down-regulated in all three NMD mutants. Of transcripts that are differentially regulated in *smg7-1* plants compared to wild-type, 54% are similarly regulated in *upf1-5* or *upf3-1* mutants. A comparable proportion of *UPF3*–regulated transcripts (47%) were commonly regulated by another NMD factor. In contrast, more than 80% of the transcripts regulated by *UPF1* also respond to another NMD factor. For both the up and down-regulated transcripts, the overlap is greater than predicted by chance (p≤0.001), as expected for independent mutants affecting the same process. Only genes that respond similarly in all three mutants were carried forward for further analyses (listed in Tables S1 and S2). Although this conservative selection of genes will almost certainly exclude some interesting results, its use ensures that genes studied here are differentially regulated by NMD, rather than unrelated processes dependent on individual NMD factors.

### There are few evolutionarily conserved targets of NMD

The 206 transcripts that are up-regulated in all three NMD mutants will be referred to as the ‘common NMD transcripts’. These include both direct targets of NMD and transcripts that are up-regulated as an indirect effect of compromising the NMD process, for example as a result of the increased stability of an mRNA encoding a transcription factor. The 132 down-regulated transcripts are presumably all indirectly affected, since targets of NMD would be expected to be exclusively up-regulated in NMD-compromised plants. All of the genes that are affected by NMD, directly or indirectly, can provide valuable clues to the biological roles of NMD-regulation of physiological transcripts. By comparing the *Arabidopsis* common NMD transcripts to the lists of genes affected by NMD in other systems, we can determine the extent to which common processes are regulated by NMD across evolution.

**Figure 2:**
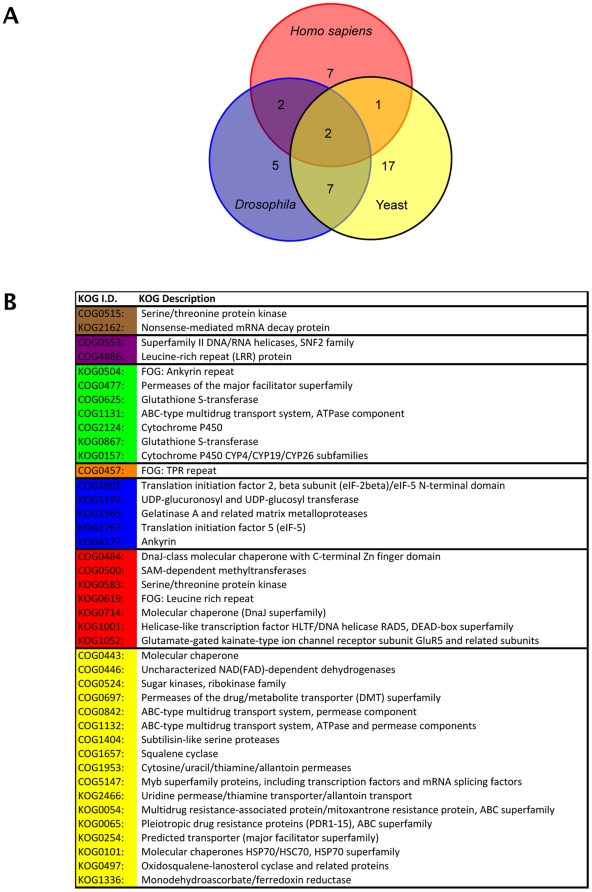
The overlap of KOGS regulated by NMD between *Arabidopsis* and other organisms. (A) A Venn diagram of the 42 *Arabidopsis (At)* NMD-regulated KOGs that are also regulated by NMD in at least one of; *Drosophila (Dm)*, *Saccharomyces cerevisiae* (Yeast, *Sc*) and Human HeLa cells (*Homo sapiens*, Hs). (B) The KOGs represented in the Venn diagram. KOGs are colour coded according to the organisms in which they are NMD-regulated: Blue: *Dm* and *At*, yellow: *Sc* and *At*, red: Hs and *At*, green: *Dm*, *Sc* and *At*, purple: *Dm*, Hs and *At*, orange *Sc*, Hs and *At* and brown: all four organisms.

Microarray analyses in *Drosophila*, humans and *S. cerevisiae* previously identified only two clusters of orthologous groups (KOGs) of genes that are regulated by NMD across kingdoms [4]. The 206 common NMD genes of *Arabidopsis* map to 165 KOGs [51]. As KOG databases are under constant revision and a further list of NMD-regulated genes is now available for yeast [5], KOGs were reassigned to NMD-regulated genes for each previously studied organism. Forty-two *Arabidopsis* common NMD KOGs overlapped with NMD-regulated KOGs of at least one other organism (Figure 2, listed in Table S3). The re-annotation of KOGs allowed us to identify an additional COG, COG0515 (serine/threonine protein kinase), as NMD-regulated in all four organisms.

One of the previously identified conserved targets, KOG2504 (monocarboxylate transporter) was not represented in the *Arabidopsis* genome. A manual inspection of the TAIR website [52] identified the product of *At2g39210*, which is a common NMD transcript up-regulated in all three NMD mutant *Arabidopsis* lines, as a putative monocarboxylate transporter of unknown function that is located in the plasma membrane. The human NMD-regulated KOG2504 gene is involved in accumulation of pyruvate in hypoxic cells [53], whereas both the yeast gene [54] and *At2g39210* [55] may be associated with nitrogen homeostasis. In summary, it is unclear whether KOG2504 represents a genuine conserved target of NMD, but the regulation of these genes by NMD may fit with the previously suggested conserved functions of NMD in amino acid homeostasis and the response to oxidative stress.

**Figure 3:**
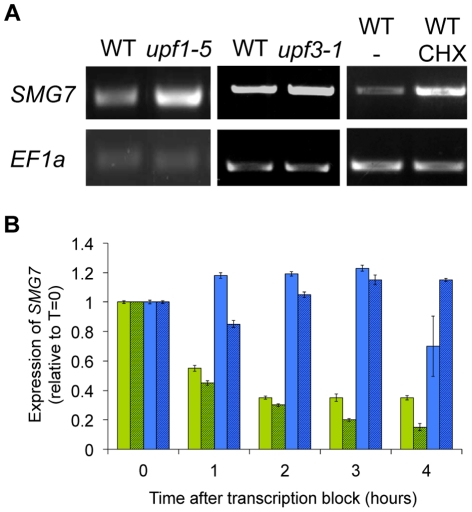
*SMG7* mRNA is stabilised in NMD mutant plants. (A) Steady state levels of *SMG7* mRNA in wild-type, *upf1-5* and *upf3-1* mutant plants and plants treated with cycloheximide to disrupt NMD, determined by reverse transcriptase PCR. (B) Degradation of *SMG7* mRNA in wild-type and *upf3-1* mutant plants, determined using real-time qPCR. The error bars represent the standard error of the mean of three technical replicates. The results of two independent treatments are shown. Green bars represent wild-type plants. Blue bars represent *upf3-1* mutant plants.

*SMG7* belongs to the other previously identified conserved target KOG: KOG2162 (nonsense-mediated decay protein). The *SMG5/7* gene, itself part of the NMD machinery, is targeted by NMD in all organisms studied [2]–[6], [9], [32], [56], implying an auto-regulatory mechanism for NMD. Previously, up-regulation of *SMG7* expression was observed in *Arabidopsis* NMD mutants and in tobacco leaves in which *UPF* genes have been silenced [9]. This study finds that *SMG7* expression is up-regulated in the *upf1-5* and *upf3-1* mutant microarray experiments and this is verified by PCR and real-time PCR (Figure 3). Furthermore, treatment with cycloheximide, which blocks translational elongation and consequently NMD, results in accumulation of *SMG7* mRNA in wild-type plants (Figure 3A). To determine whether *SMG7* is a direct target of NMD, cordycepin was used to halt transcription and the rate of decay of *SMG7* mRNA in wild-type and *upf3-1* mutant seedlings was compared by real-time PCR (Figure 3B). The rate of decay of *SMG7* mRNA is higher in wild-type plants than in *upf3-1* mutants, showing that the disruption of *UPF3* stabilises *SMG7* mRNA. This validates our microarray and provides compelling evidence that *Arabidopsis SMG7* is a direct target of NMD.

It has been noted that a surprising number of NMD-regulated genes in yeast are associated with the dynamics of the plasma membrane and cell wall, particularly with respect to multi-drug resistance [5]. This study identifies a number of similar KOGs that are NMD regulated in *Arabidopsis* (Figure 2 and Table S3). Eight KOGs are represented amongst NMD-regulated genes in both *Arabidopsis* and yeast: COG0697 (permeases of the drug/metabolite transporter (DMT) superfamily); COG0842 (ABC-type multidrug transport system, permease component); COG1132 (ABC-type multidrug transport system, ATPase and permease components); KOG0054 (multidrug resistance-associated protein/mitoxantrone resistance protein, ABC superfamily); KOG0065 (pleiotropic drug resistance proteins (PDR1-15), ABC superfamily), KOG0254 (predicted transporter (major facilitator superfamily)), COG0477 (permeases of the major facilitator superfamily) and COG1131 (ABC-type multidrug transport system, ATPase component). The latter two are also regulated by NMD in *Drosophila*. This suggests that many multi-drug transporters are regulated by NMD in diverse organisms.

**Figure 4:**
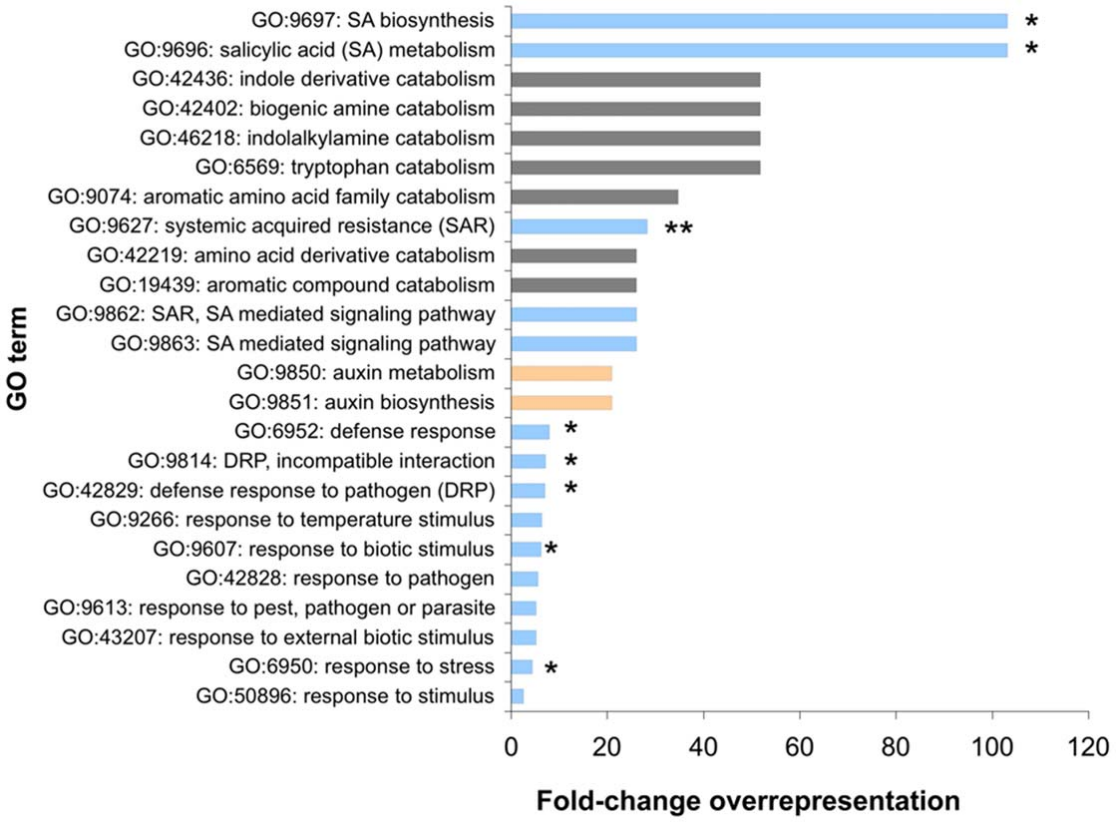
Biological function of NMD-regulated genes. Biological function gene ontology (GO) terms that are over-represented (p<0.05) amongst the 206 genes that are commonly up-regulated in NMD mutant plants. The horizontal bar indicates the proportion of the 206 NMD genes with the given GO term relative to the proportion of the genome with the same GO term. DRP is defence response to pathogen, SA is salicylic acid and SAR is systemic acquired resistance. GO terms relating to response to stimuli are coloured blue. GO terms relating to auxin metabolism are coloured peach. The remainder are all associated with amino acid catabolism. * p value≤0.01. ** p value≤0.001.

Although evolutionarily common NMD target genes have been difficult to identify [4], there is evidence to suggest that certain biological processes are conserved under the control of NMD in different kingdoms. These processes include amino acid homeostasis [3], [4], [35], [57] and protection from oxidative stress [34]. Transcripts encoding proteins involved in amino acid metabolism are overrepresented amongst human NMD targets [3] and present (though not overrepresented) amongst *S. cerevisiae* NMD targets [2], prompting the hypothesis that NMD has a conserved role in regulating the expression of genes involved in amino acid homeostasis. This hypothesis was further supported by similarities between the gene expression profiles of NMD impaired *Drosophila* cells and wild-type starved *Drosophila* [4]. The common NMD transcripts in *Arabidopsis* are enriched for genes involved in the catabolism of amino acids and their derivatives (Figure 4). Of the 16 common NMD genes that have GO terms implicating them in amino-acid homeostasis, ten have features that could target them to NMD; eight have upstream open reading-frames (uORFs), one has a 3′UTR in excess of 300 nt and one has both of these features. This enrichment of NMD target features may imply that this functional sub-set of NMD-regulated genes includes direct targets of NMD. Taken together, these results suggest that in *Arabidopsis*, as in other organisms, NMD is involved in amino-acid homeostasis.

### Pathogen response transcripts are up-regulated in *Arabidopsis* NMD mutants

Amongst the common NMD genes, there is a dramatic overrepresentation of transcripts encoding proteins involved in responses to pathogens (Figure 4). Strikingly, all of the functional ontologies that are overrepresented with a p value of <0.01 are related to pathogen response, including defence response (31 genes), systemic acquired resistance (5 genes) and defence response, incompatible interaction (8 genes). The list includes known pathogen induced genes including the key regulators of salicylic acid (SA) mediated plant defence *EDS1*, *EDS5* (*sid1*), *ICS1* and the classical molecular markers of SA activated plant defence *PR1* and *PR5* (reviewed in [58]). Genes involved in indole biosynthesis and more specifically auxin biosynthesis are overrepresented (p<0.05). Notably, other stress-responsive transcripts, such as those involved in the responses to wounding or oxidative or osmotic stress, were not overrepresented. Taken together, these observations imply that these transcriptional differences are not a general stress response resulting from a deleterious mutation, but a direct result of deficient NMD leading to the up-regulation of pathogen-responses involving the SA signalling pathway. Many pathogen responses result from co-ordinate regulation by SA, JA and ethylene [59], [60], yet no GO terms related to JA or ethylene were over-represented amongst the common NMD genes. This suggests that neither the impairment of NMD, nor the consequent overproduction of salicylic acid, leads to ethylene or JA-dependent responses in *Arabidopsis*.

**Figure 5:**
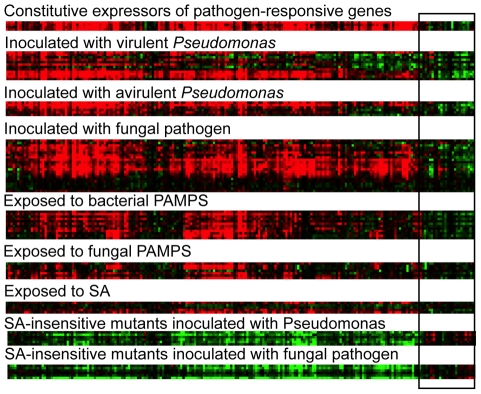
The expression of the 206 ‘common NMD genes’ in response to biotic stimuli. Green indicates that genes are down-regulated, red indicates up-regulation and black indicates that there was no change. The box surrounds genes that were not pathogen-responsive.

As there was an overrepresentation of gene ontologies relating to pathogen responses amongst the common NMD genes, publicly available microarray data were mined for expression levels of all of the common NMD genes in response to pathogens, pathogen-associated molecular patterns and SA (Figure 5). The profiles fitted two broad clusters, with 183 of the 206 common NMD genes forming a group of pathogen-responsive genes and 23 common NMD genes being unresponsive (listed in Table S4). All of the pathogen-responsive genes were down-regulated in SA insensitive mutant plants, suggesting a dependency on the SA signalling pathway (Figure 5). Closer inspection of the SA and auxin biosynthetic pathways revealed that five common NMD genes are involved in the utilisation of chorismate to produce camalexin and SA or to yield tryptophan, the precursor for the synthesis of auxin. Other genes in these pathways were also up-regulated in NMD mutants, but had not passed the rigorous selection criteria (up-regulated at least 1.5 fold at p≤0.05 in each of the three mutants, Table S5). Genes involved in auxin biosynthesis respond systemically [61] and locally to infection with *P. syringae* [43], [62]. Of the 12 genes involved in auxin synthesis that are up-regulated in at least one of the NMD mutant lines, six are also associated with GO terms linked to defence responses. We therefore suspect that the overrepresentation of genes involved in auxin metabolism is part of the SA response rather than a direct result of regulation of auxin metabolism by NMD.

### NMD mutants are partially resistant to *Pseudomonas syringae* DC3000

**Figure 6:**
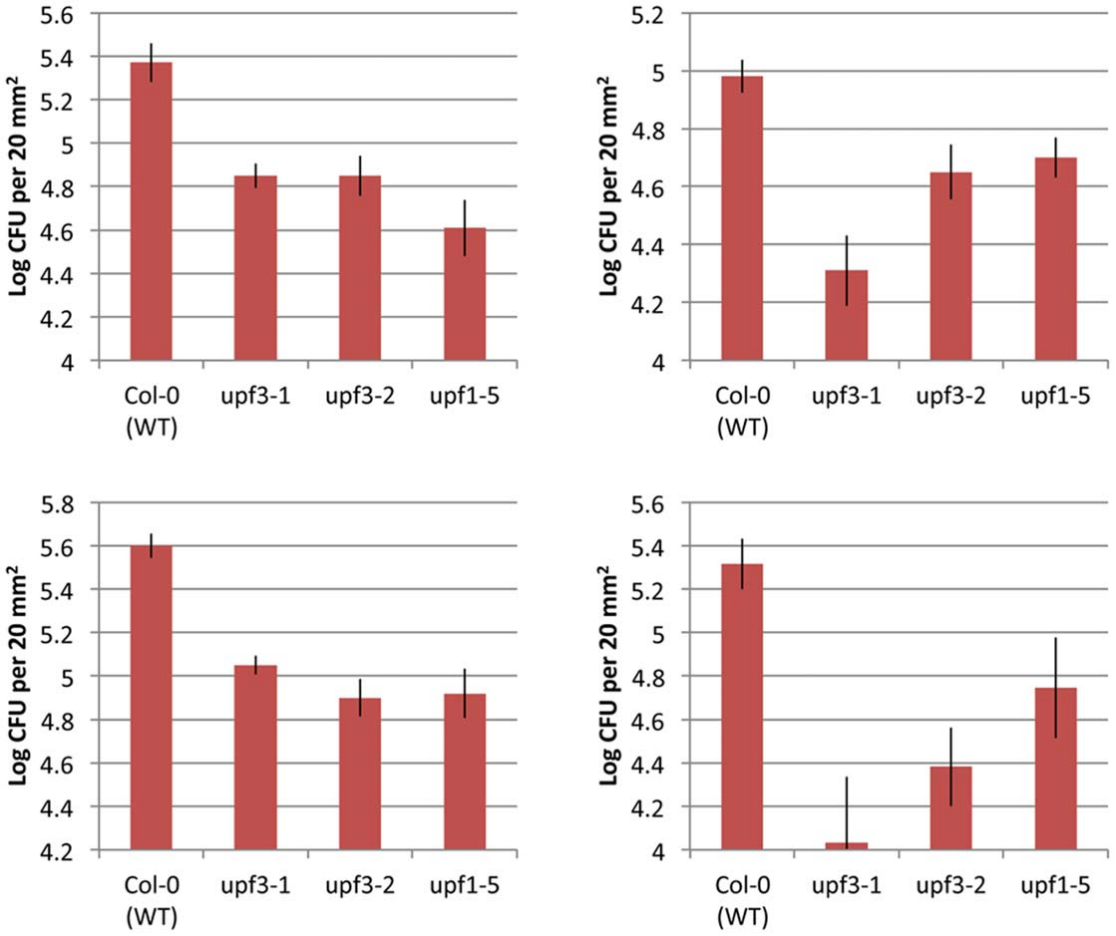
Reduced growth of *Pseudomonas syringae* DC3000 in NMD mutants. Growth of *Pseudomonas syringae* DC3000 in wild-type and NMD mutant *Arabidopsis* leaves. Numbers of colony forming units were counted 3 days post infection. Bacterial counts are on a log_10_ scale. Results from independent replicate experiments are shown.

The speed with which plants respond to pathogenic attack by up-regulating inducible defence genes is a key factor in determining resistance or susceptibility [63]. Given the strong correlation to transcriptional reprogramming in response to infection with the hemibiotroph *Pseudomonas syringae* pathovar tomato DC3000 (DC3000) [43], [62], we hypothesised that the constitutive up-regulation of pathogen-responsive genes observed in NMD-mutant plants may confer resistance to pathogens. *upf1-5*, *upf3-1* and *upf3-2* mutant plants were challenged with virulent DC3000, however the severe effect of the mutation on the width and shape of the leaf blade prevented us from assaying *smg7-1* plants. Significantly fewer colony-forming units were isolated from leaves of DC3000 challenged *upf1-5*, *upf3-1* and *upf3-2* mutant plants than the wild-type control 3 days post-inoculation (Figure 6). This suggests that NMD mutant plants are more resistant to DC3000.

**Figure 7:**
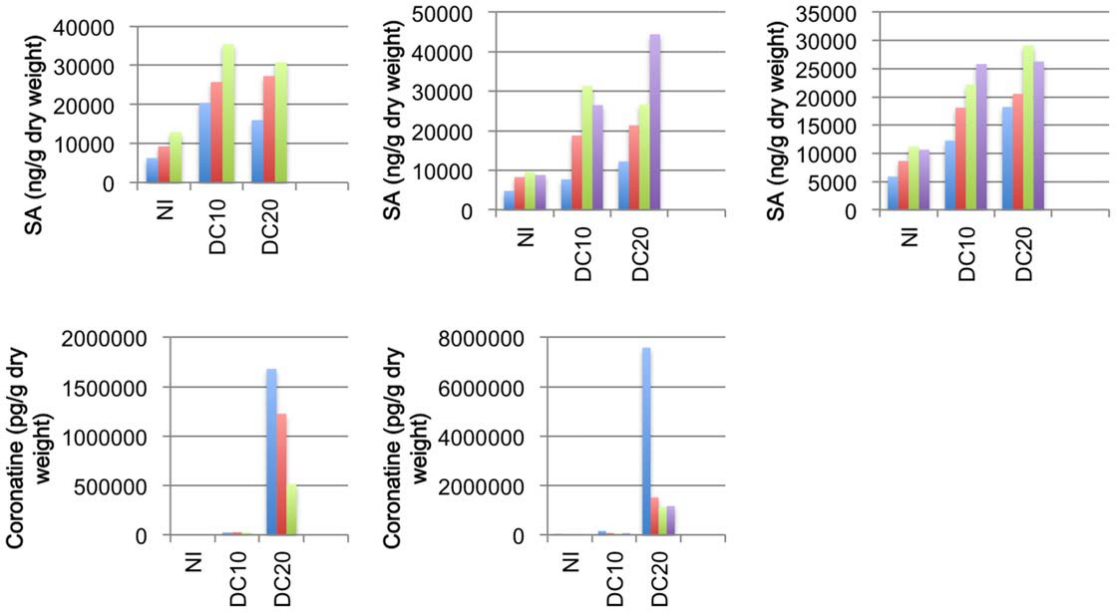
Levels of salicylic acid and coronatine in NMD mutants. Salicylic acid (SA) content and coronatine content of wild-type and NMD mutant plants. The salicylic acid and coronatine levels of leaves were measured prior to inoculation (NI) and 10 or 20 hours post-infection with *Pseudomonas syringae* DC3000 (DC10 and DC20 respectively). Wild-type plants are represented by blue bars, *upf1-5* mutants by red bars, *upf3-1* by green bars and *upf3-2* plants by purple bars.

Pathogen induced hormonal imbalances underpin many pathogen responses [64], [65]. SA is primarily associated with defence against biotrophic pathogens and the activation of SAR [48], [49]. ABA promotes virulence of diverse pathogens against various host species by down-regulation of *ISOCHORISMATE SYNTHASE 1* (*ICS1*) expression, which is required for SA production, and suppression of PAMP-induced transcripts (reviewed in [66]). Upon infection of *Arabidopsis* with compatible DC3000, coronatine, SA and ABA accumulate rapidly, wheras JA production is induced later in the infection process [50]. The hormone levels of wild-type and mutant plants were profiled before and during infection with DC3000. While levels of JA and abscisic acid (ABA) do not vary significantly between genotypes (p>0.05 in at least 2 of triplicate experiments, Figure S1), levels of SA are significantly higher in NMD mutant plants than in wild-type, even prior to infection (p<0.05 in at least 2 of triplicate experiments, Figure 7). In accordance with the reduced bacterial load, levels of the Pseudomonad phytotoxin coronatine [65] are lower in infected NMD mutant plants compared to wild-type (Figure 7). This suggests that the up-regulation of pathogen responsive genes in NMD mutant *Arabidopsis* plants, and their enhanced resistance to DC3000, is a result of constitutive activation of the SA synthesis pathway.

### CuORFs are massively enriched amongst the NMD targets that are not pathogen responsive

Several gene features have been associated with NMD substrates in a range of different model systems. In plants, these include upstream open reading frames (uORFs) [67], UTRs harbouring an intron and 3′UTRs in excess of 300 nt [68]. In common with most NMD target gene analyses in other systems [2]–[4], there was no convincing enrichment of any of these features amongst the common NMD genes in *Arabidopsis* (Table S6). For example, 34% of the 206 common NMD genes encode transcripts with an upstream open reading frame (uORF), in comparison to 30% of all *Arabidopsis* genes [69]. Limiting these analyses to the 23 non-pathogen responsive genes allows us to exclude the effects of activation of the SA-mediated defence response in up-regulating genes that are not directly targeted by NMD. Amongst these 23 genes, uORFs and 5′UTR introns are over-represented by more than two-fold. Surprisingly, one feature was dramatically over-represented amongst a sub-set of the common NMD genes. Conserved peptide uORFs (CuORFs) are uORFs encoding evolutionarily conserved amino acid sequences. Forty-four *Arabidopsis* genes with CuORFs have been previously identified by comparisons of *Arabidopsis* and rice full length cDNAs [70]. Transcripts with CuORFs are 21-fold over-represented amongst common NMD genes (Table S4, 8 transcripts, p<0.0001). In contrast, no CuORF-containing genes were commonly down-regulated in the NMD mutants. However, when the NMD-regulated CuORFs were mapped onto the cluster analysis, it became clear that all of them belong to the group of 23 common NMD genes that do not respond to pathogens, representing a 190-fold enrichment for this feature.

## Discussion

### NMD influences the expression of many *Arabidopsis* genes

The 338 *Arabidopsis* genes (1.6% of transcripts represented on the array) that are either up- or down-regulated in all three mutant backgrounds tested represent a very conservative list of transcripts that are directly or indirectly controlled by NMD in wild type *Arabidopsis* plants. The fact that the NMD mutants used are unlikely to be null, the stringency of the microarray analyses and the use of a developmentally restricted stage, suggests that many more *Arabidopsis* transcripts are affected by NMD. Similar results have been reported in *S. cerevisiae* [2], [5], humans [3], *Drosophila* [4] and *Caenorhabditis elegans* [71], where estimates of the proportion of physiological mRNA species affected by NMD range from 3% to greater than 10%. It therefore seems in plants, as in other systems, that the abundance of large numbers of ‘normal’ physiological transcripts is influenced positively or negatively by NMD. However, it is still not clear what proportion of the transcripts influenced by NMD represent direct targets of the process. A microarray mRNA stability analysis suggested that as many as 45% of the *S. cerevisiae* mRNAs regulated by NMD represent direct targets [5], whereas other analyses in human cell cultures have suggested that the direct targets of NMD represent a smaller proportion of the overall numbers of affected transcripts [72], [73]. Further work will be required to identify the true proportion of direct NMD target transcripts in different systems and such analyses should also help to define the typical features that identify direct targets of NMD amongst physiological transcripts.

Our microarray results are compatible with previous analyses of gene expression in other alleles, using different platforms and under different growth conditions [6], [74], with overlaps of 29% and 52% respectively. The significant overlaps between different experimental platforms and growth conditions, as well as between different mutant alleles, validate the microarrays and confirm the identification of large numbers of NMD-regulated transcripts in *Arabidopsis*.

### Evolutionary conservation of NMD target genes

It has previously been noted that few orthologous genes are commonly regulated by NMD across diverse organisms [4]. The fresh analyses of NMD-regulated genes from *Drosophila*, yeast and human cells, coupled with the *Arabidopsis* NMD-regulated KOGs supports this, with only two KOGs commonly up-regulated across all four organisms. Homologues of the *SMG5-7* genes, represented in KOG2162 (NMD protein), are direct targets of NMD in all organisms tested [2]–[6], [32], [56]. It appears that the auto-regulation of these genes is a conserved mechanism for the control of NMD. COG0515 (serine/threonine protein kinase) was also regulated by NMD in all four organisms.

Multidrug resistance may represent another common process affected by NMD. Genes involved in multidrug transport are suppressed by NMD in yeast, *Arabidopsis* and *Drosophila*. Although this study did not identify KOGs containing multidrug transporters amongst the human NMD targets, there is a known example. MRP4 is a mammalian cellular efflux pump. An MRP4 cDNA identified from humans contains two additional exons, which introduce a premature termination codon. These exons are highly conserved across humans, rodents and monkeys and appear to target the mRNAs that harbour them to NMD [75]. Involvement of NMD in the regulation of multi-drug resistance is intriguing, as transporters tend to be expressed specifically in response to their substrates. This implies that the regulation of such genes by NMD would need to be both highly specific and adaptive.

Molecular chaperones were well represented amongst KOGs that are NMD-sensitive in *Arabidopsis* and at least one other organism: COG0443 (yeast, molecular chaperone); KOG0101 (yeast, molecular chaperones HSP70/HSC70, HSP70 superfamily) and KOG0714 (human, molecular chaperone (DnaJ superfamily)). Abrogation of NMD is expected to result in an increased number of mRNAs encoding truncated proteins. If translated, these may induce the unfolded protein response and account for the up-regulation of genes encoding molecular chaperones.

KOGs up-regulated in both *Arabidopsis* and *Drosophila* include COG1601 (translation initiation factor 2, beta subunit (eIF-2beta)/eIF-5 N-terminal domain) and KOG2767 (translation initiation factor 5 (eIF-5)). In human cells, phosphorylation of eIF-2 results in the localisation of UPF1/RENT1 in stress bodies, which inhibits NMD [34]. EIF-5 is involved in recycling of eIF-2 to its active form [76]. Perhaps the suppression of *eIF-2* and *eIF-5* expression by NMD represents another mechanism, in addition to regulation of *SMG5-7* homologues and phosphorylation state of eIF-2alpha, by which NMD is auto-regulated.

NMD regulates gene expression in all organisms tested [2]–[4], [6], [71] and it is, therefore, surprising that there appear to be so few evolutionarily conserved targets of this ancient mechanism. It is possible that roles for NMD in addition to transcript quality control, evolved independently in diverse organisms. It seems likely however that the use of different experimental techniques and growth conditions, as well as conservative lists of potential NMD targets and limited KOG annotations, have impeded the identification of conserved targets.

### A biological rationale for NMD in plants

NMD-mutant *Arabidopsis* plants constitutively express pathogen-responsive genes and have higher levels of SA than wild-type plants, even in the absence of pathogens. This impairs the virulence of DC3000 on NMD-mutant plants. The possibility remains that the SA pathway is activated in response to the general stress caused by the accumulation of erroneous transcripts, however the specificity of the responsive genes suggests that this is highly unlikely. The phenotype of the NMD mutants mimics that of other *Arabidopsis* lines that overexpress pathogen responsive genes, including plants in which *R* genes are constitutively active [77], [78]. As activation of numerous *R* genes causes SA accumulation and numerous *R* genes are up-regulated in response to SA [79], [80], it is difficult to conclude whether individual NMD repressed genes are direct or indirect targets of NMD. To understand this further requires the identification of transcripts that are direct targets of plant NMD.

The *R* gene *SNC1* (*SUPPRESSOR OF NPR1-1, CONSTITUTIVE 1*) was previously shown to be up-regulated in non-inoculated *upf1-5* and *upf3-1* plants [78]. The abundance of different splice variants, relative to each other, does not appear to vary between NMD mutant and wild-type plants [78]. Unless there is another, previously undetected, splice variant of *SNC1*, this suggests that *SNC1* is unlikely to be a direct target of NMD. Our arrays found a small but reproducible up-regulation of *SNC1* in *smg7-1* plants (fold change = 1.4, p≤0.05), but not in *upf1-5* or *upf3-1*. As *SNC1* is regulated by a positive amplification loop involving SA, up-regulation of *SNC1* may be a consequence of, rather than the initial stimulus for, the accumulation of SA in NMD mutant plants.

The observation that pathogen responses are de-repressed in NMD mutant plants raises the question of whether this results from a housekeeping function of NMD, preventing the erroneous expression of aberrant but still functional *R* genes, or whether NMD is involved in the response to pathogens. *R* genes are extensively alternatively spliced and regulation of *R* gene splicing is critical for plant immunity [81]–[83]. It is probable that NMD targets splice variants that contain premature termination codons. If *R* gene splice variants containing premature termination codons are created in the absence of pathogens, these transcripts could accumulate in NMD mutant plants, leading to mis-expression of *R* genes. The generation of *R* gene splice variants that are targeted by NMD could also provide an opportunity for NMD-mediated regulation of plant-pathogen responses. Levels of NMD activity could change in response to pathogens, perhaps by sequestering one or more NMD factors as in mammalian cells under oxidative stress, or the production of transcripts could shift in favour of splice variants with or without NMD targeted features. This would provide a mechanism by which pathogen-responsive NMD targets could be removed from the sphere of influence of NMD upon infection. There is a precedent for the temporal and spatial regulation of NMD; in mice a brain-specific microRNA, miR-128, represses NMD in differentiating neuronal cells and during brain development leading to the up-regulation of NMD targeted genes that are important for normal neuronal development and function [36]. Global studies of the impact of NMD on relative rates of mRNA decay before and after infection are required to answer these important questions.

Amino-acid homeostasis has previously been suggested as a potential conserved role of NMD [3], [4]. In *Arabidopsis*, there is a known link between the regulation of genes involved in amino acid homeostasis and the pathogen response [84], [85]. Indeed, of the 16 common NMD genes that are implicated in amino acid homeostasis, 15 are in the pathogen-responsive clade of Figure 5 and seven have GO terms directly related to pathogen responses. It is possible that the role of NMD in regulation of pathogen responses has its evolutionary origin in the NMD-mediated control of homeostasis.

As sessile organisms, plants are arguably more dependent on rapid changes in gene expression in response to biotic and abiotic stressors. This is reflected in the aggressive nature of the hypersensitive response, in which localised programmed cell death restricts pathogen ingress (reviewed in [86]). The tight regulation of such responses is paramount, as the need to thwart pathogens is countered by the detrimental effects of inappropriate expression of pathogen-responsive genes [87]. Indeed, it is possible that many of the deleterious effects of NMD deficiency in *Arabidopsis* are caused by the unwarranted pathogen response. Constitutive expression of pathogen responsive genes often results in reduced fitness [87]–[89]. Reported phenotypes of *Arabidopsis* NMD mutants include spontaneous necrosis [32], small or narrow leaves that are slightly indented or jagged and impaired flowering [29]. Numerous other *Arabidopsis* mutants that overexpress SA dependent defence responses display similar spontaneous necrotic lesions [90]–[95] and effects on leaf size [96] and shape [97] have also been identified. SA is also involved in the regulation of flowering [89]. It is tempting to speculate that NMD-mediated repression of pathogen responsive processes evolved to prevent inappropriate expression of genes that have significant fitness costs. Co-ordinate repression of pathogen responses by NMD may also provide the plant with a rapid mechanism to induce pathogen responses by the stabilisation of existing transcripts.

### CuORFs are targets of NMD in *Arabidopsis*

It has long been known that the presence of uORFs in naturally-occurring transcripts can target them to NMD [98]. The identification of candidate direct NMD targets in yeast indicated that around 35% could be attributed to the presence of an uORF [5], whereas amongst 17 human direct NMD targets identified using a proteomic approach, 11 contained an uORF [73]. However, uORFs appear to be more common in the genome than direct targets of NMD, meaning that not all uORFs act as targets for NMD. Indeed, amongst our common NMD genes we did not observe a significant enrichment of uORF-containing transcripts, probably because this list contains a mixture of direct and indirect NMD targets, complicating the identification of specific NMD target signals. Similarly, only a subset of uORF-containing transcripts in *C. elegans* is elevated in an NMD mutant background [71] and no clear defining feature of the uORFs associated with NMD-targeted transcripts could be identified amongst the 11 human NMD target genes [73].

Although uORFs in general did not stand out from the background as a putative NMD targeted feature in our microarray analysis, one particular subset of uORFs is enhanced in the common NMD genes. Evolutionarily conserved uORFs (CuORFs) have been described following a comparison of cDNAs from *Arabidopsis* and rice [70]. Forty-four such genes, belonging to 19 homology groups, were identified from a comparison of *Arabidopsis* and rice cDNA sequences. Eight of these CuORFs are present in the common NMD gene list (8/206 belonging to homology groups 1, 4, 7, 10, 13, 15 and 17 [70]) whereas none are present in the list of transcripts down-regulated in all three mutants (0/132). Even more strikingly, all 8 of the CuORFs found in the common NMD list belong to the sub-group of 23 NMD target genes that are not responsive to pathogens (Table S4), representing an almost 200-fold enrichment. This finding strongly suggests that CuORFs are substrates of NMD in *Arabidopsis* and, given their levels of conservation, potentially in other plant species.

It is not obvious why CuORFs should differ from other uORFs in their ability to target a transcript to NMD. In tobacco, the observation that an uORF of 153 nt was targeted by NMD, whereas those of 108 or less did not, led to the hypothesis that plant NMD targets uORFs that are translatable and encode a peptide of at least 53 amino acids; 331 *Arabidopsis* genes satisfy these criteria [67]. Of the eight NMD responsive CuORF-containing genes identified here, two have CuORFs that encode peptides of less than 35 amino acids, five have CuORFs that encode peptides of 41–52 amino acids and one has a CuORF that encodes a peptide of 57 amino acids. It is unlikely, therefore, that uORF length alone accounts for the overrepresentation of CuORFs amongst the NMD targets. Re-initiation of translation downstream of a premature termination codon can protect an mRNA from NMD [99]. An intriguing possibility is that the conserved peptides produced by these transcripts act, potentially in a condition-dependent fashion, either to decrease the efficiency of the re-initiation of translation at the major (downstream) AUG, or stall the ribosome at the CuORF, thereby activating the NMD pathway to degrade the mRNA. The latter mechanism has already been described in *S. cerevisiae* for *CPA1*, which is targeted by NMD in response to arginine. The uORF of *CPA1* encodes arginine attenuator peptide (AAP). In the presence of arginine, ribosomes are stalled at the uORF. The resultant increased association of ribosomes with the uORF and decreased association of ribosomes with the major ORF targets the transcript to NMD [57], [100]. Either of these mechanisms would allow a constant rate of NMD to play a role in the control of individual genes by effectively using the conserved peptides to move specific transcripts into and out of the sphere of influence of NMD. Most of the CuORF genes in the common NMD list are currently uncharacterised, but one of them, *SUPRESSOR OF ACAULIS51* (*SAC51*), encodes a bHLH transcription factor that has been shown to regulate the *ACL5* gene, which encodes thermospermine synthase [101], [102]. Consistent with the role of NMD in suppressing *SAC51* transcript levels using a CuORF, the *sac* mutant, in which *SAC* expression is elevated, is caused by a point mutation in the CuORF [103]. The group of CuORF-containing transcripts forms a candidate list for individual genes regulated by NMD under diverse conditions and it will be interesting to see whether other CuORF-containing genes, not identified as NMD targets under our conditions, are regulated by NMD at different stages of development or under different conditions. A role for NMD in targeting transcripts with CuORFs could have regulatory effects extending far beyond the CuORF-containing genes, as the overrepresentation of transcription factors amongst CuORF-containing genes suggests that the expression of numerous genes could be affected indirectly by NMD.

In conclusion, we suggest that conserved upstream open reading frames represent specific targets of NMD and may imply a mechanism for the differential regulation of subsets of NMD targets in plants. We also present a biological rationale for the co-ordinate regulation of non-aberrant plant genes by NMD. We show that NMD represses expression of genes involved in the SA-mediated pathogen response and that lifting this repression, by abrogation of NMD, confers partial resistance to *Pseudomonas syringae* pathover tomato DC3000. This raises the possibility that plants may regulate NMD as a means to coordinate pathogen responses. *Pseudomonas syringae* causes a range of diseases to numerous agronomically important crops [100]. Although we have not exposed the NMD mutants to other pathogens, it seems likely that the constitutive up-regulation of pathogen response genes observed may also confer resistance to numerous other pathogens. Perhaps in the future an understanding of the interplay between plant pathogen responses and NMD will facilitate the development of novel approaches in crop protection.

## Materials and Methods

### Plant materials and growth conditions

Transgenic plants were obtained from the Nottingham *Arabidopsis* Stock Centre (NASC) in the UK [104]. For microarray analyses, wild-type (ecotype Columbia-0) and T-DNA insertion lines SALK_112922 (*upf1-5*), SALK_073354 (*smg7-1*) and SALK_025175 (*upf3-1*) were grown on 3∶1 SHL soil∶sand (22–24°C, under constant light). For qPCR and experiments involving *Pseudomonas*, plants were grown under 8 hours light (175 µ mol m^−2^ s−^1^)/16 hours dark at 65% relative humidity in SHL compost.

### Microarray hybridisation

RNA was isolated from 17 day-old seedlings. Approximately 5 µg of total RNA from each sample was used to produce cDNA using the GeneChip® One-cycle cDNA synthesis kit (Affymetrix), as per manufacturer's instructions. Double stranded cDNA products were purified using the GeneChip® Sample Cleanup Module (Affymetrix). The synthesised cDNAs were in-vitro transcribed by T7 RNA polymerase using biotinylated nucleotides to generate biotinylated complementary RNAs (cRNAs) using the GeneChip® HT IVT labeling kit (Affymetrix), as per manufacturer's instructions. The cRNAs were purified using the GeneChip® Sample Cleanup Module (Affymetrix). The cRNAs were then randomly fragmented at 94°C for 35 minutes in a buffer containing 40 mM Tris-acetate (pH 8.1), 100 mM potassium acetate, and 30 mM magnesium acetate to generate molecules of approximately 35 to 200 bp. Affymetrix *A. thaliana* ATH1-121501 GeneChip® arrays were hybridised with 15 µg of fragmented labelled cRNA for 16 h at 45°C as described in the Affymetrix Technical Analysis Manual using the GeneChip® hybridization control kit and GeneChip® hybridisation, wash and stain kit (Affymetrix). GeneChip® arrays were stained with streptavidin-phycoerythrin solution and scanned with an Affymetrix 3000 7G GeneArray scanner. Following scanning, non-scaled RNA signal intensity (CEL) files were generated using GeneChip® operating software (GCOS; Affymetrix). All data is MIAME compliant and the raw data has been deposited in both ArrayExpress (E-GEOD-19253 and E-GEOD-32671) and GEO (GSE19253 and GSE32671). The raw data is also available from the NASCArrays database (NASCARRAYS-379 and NASCARRAYS-418, http://affymetrix.Arabidopsis.info/; [105]).

### Microarray analyses

The non-scaled RNA CEL files were loaded into GeneSpring analysis software (GeneSpring 7.3; Agilent Technologies, USA) using the Robust Multichip Average (RMA) pre-normalisation algorithm [106]. Separate experiments were created for each mutant (*upf1-5*, *upf3-1* and *smg7-1*) using the normalised CEL files for the mutant and corresponding wild-type RNAs. Further normalisations were performed for each experiment using a three step process: (i) probe-sets with a signal value <0.01 were set to 0.01, (ii) per chip normalisation to the 50th percentile, (iii) each gene signal was normalised to the median of that gene. Putative genes with differential hybridisation intensities between a single mutant and corresponding wild-type were identified using a two-step process: (i) genes that were 1.5-fold up- or down-regulated were selected, and (ii) a Welch's t-test was performed (p<0.05). A condition tree was generated for these genes using K-means clustering across conditions (*Arabidopsis* lines) in GeneSpring. In brief, hierarchical clustering was used to compare the similarity in the expression profiles of this group of genes between *Arabidopsis* lines. Genes that were differentially expressed in more than 1 mutant were identified by comparing gene lists of differentially expressed genes in a single mutant using the Venn diagram function of GeneSpring. The DAVID Gene Functional Classification Tool [107], [108] was used to search for ontologies that were over-represented amongst the commonly up- or down-regulated NMD genes. Java Treeview [109] was used to profile the expression of the common NMD genes across publicly available microarray data. The STRING database [51] was used to assign KOGs to NMD regulated genes from *Arabidopsis* and other organisms. To create Figure 5 selected publicly available experiments (Table S7) were processed using Bioconductor to yield gcrma normalised data. Replicates were averaged and the log (base2) ratio to control treatments calculated. The arrays were left as ordered but the genes were organised by SOM and then clustered using hierarchical clustering by uncentered correlation and complete linkage.

### Cycloheximide treatment

Leaves of soil-grown wild-type and mutant plants were collected into 2-ml Eppendorf tubes containing MS medium with or without 20 µM cycloheximide and incubated at room temperature for 15 minutes. Following vacuum infiltration for five minutes, the samples were incubated for three hours and then frozen for RNA extraction.

### Cordycepin treatment

Wild-type and *upf3-1* plants were grown on MS plates for two weeks. Plants were then transferred to flasks containing incubation buffer (1 mM Pipes, 1 mM sodium citrate, 1 mM KCl, 15 mM sucrose). After 30 minutes, all seedlings except the time = 0 samples were transferred to fresh flasks containing incubation buffer plus cordycepin at 150 µg/ml. All flasks were then subject to a short (1–2 minute) vacuum infiltration. Time = 0 samples were immediately frozen in liquid nitrogen. 4–5 seedlings per replicate per time-point were taken from the cordycepin-containing flasks and frozen, every hour for four hours.

### RNA extraction, RT-PCR and qPCR analyses

Tissue was harvested and snap frozen in liquid nitrogen. Total RNA was extracted from leaves or whole plants using the QIAGEN RNeasy kit as per manufacturer's instructions, including the optional on-column digestion of DNA. mRNA levels were examined by RT-PCR and two micrograms of total, DNA-free RNA were reverse transcribed using Superscript II MMLV Reverse Transcriptase. RNAse H was used to destroy any residual RNA and 2 µl of the reaction were then used as a template in a PCR as follows: an initial cycle at 95°C for 3 min followed by 27–37 cycles at 95°C for 1 min, 55–65°C (depending on Tm of primers) for 1 min and 72°C for 3 min, and a final cycle at 72°C for 5 min. The number of cycles was adjusted in each case to avoid over-cycling and all RT-PCR assays were carried out in duplicate. Selected RT-PCR assays were confirmed by qPCR. qPCR was carried out in the presence of the SYBR Green I dye, which binds to double stranded DNA and was monitored with the iCycler thermal cycler (BIO-RAD). Relative values of target expression were calculated as the average of two biological and three technical replicates by normalisation to the EF1α control. Primer sequences are given in Table S8.

### Pseudomonas growth assays

A scalpel was used to nick the underside of fully expanded leaves of 5–6 week old plants either side of the mid-vein. A needleless syringe was then used to infiltrate leaves with DC3000 (A_600_ = 0.0002) suspended in 10 mM magnesium chloride. 3 days post-infection, a cork borer was used to excise a disc from the infection site. Bacteria were extracted by homogenisation in 10 mM magnesium chloride and cultured on Kings B medium at 28°C. The next day, the numbers of colony forming units were counted. Each experiment consisted of 3 leaves each from 4–6 plants of each genotype.

### Hormone profiles

Post-inoculation with DC3000 (A_600_ = 0.15), plants were grown under constant light for 10 or 20 hours prior to harvest of inoculated leaves. Each wild-type sample consisted of three leaves from each of two plants. Due to their smaller size, four leaves from each of three mutant plants were pooled/sample. For each time-point, triplicate samples were collected and the mean and SEM were calculated. At harvest, samples were immediately snap frozen in liquid nitrogen. The experiment was performed three times. Hormone measurements were carried out as described in [110].

## Supporting Information

Figure S1 **Levels of jasmonic acid and abscisic acid in freeze dried tissues of wild type and NMD-deficient** ***Arabidopsis*** **leaves.** Leaves were harvested from non-inoculated (NI) plants and plants 10 and 20 hours after infection with *Pseudomonas syringae* DC3000 (DC10 and DC20). Wild-type plants are represented by blue bars, *upf1-5* mutants by red bars, *upf3-1* by green bars and *upf3-2* plants by purple bars.
(TIFF)

Table S1 **Genes that are co-ordinately up-regulated in NMD mutant** ***Arabidopsis*** **(common NMD genes).**
(XLS)

Table S2 **Genes that are co-ordinately down-regulated in NMD mutant** ***Arabidopsis*** **.**
(XLS)

Table S3 **KOGs represented by** ***Arabidopsis*** **common NMD genes.** The shading indicates the number of species for which genes belonging to the given KOG are up-regulated in response to abrogation of NMD. Darker shades indicate a greater number of species.
(XLS)

Table S4 **23 common NMD genes that are not pathogen responsive, those with a CuORF are highlighted.**
(XLS)

Table S5 **Genes that are both regulated by NMD factors and have a role in auxin synthesis, or in the utilisation of chorismate.** Where a gene is commonly up-regulated in all three NMD mutant lines it is shaded red. Orange indicates up-regulation in at least two NMD mutant lines and yellow indicates up-regulation in only one. Genes that are down-regulated in a mutant line are shaded light green. Those genes commonly down-regulated in two NMD mutant lines are coloured dark green.
(XLS)

Table S6 **Putative NMD-targeted features of the common NMD genes.** The 23 genes that are not in the pathogen-responsive clade are highlighted.
(XLSX)

Table S7 **Microarray experiments represented in** **Figure 5** **.** Four databases were used to compile Figure 5. The identifiers for the experiments used are given beneath the url for the database from which they were retrieved.
(XLSX)

Table S8 **Primers used in this study.**
(XLS)
