# Supplementary figures and images for: A Role for Nonsense-Mediated mRNA Decay in Plants: Pathogen Responses Are Induced in Arabidopsis thaliana NMD Mutants
Source: PLoS One. 2012 Feb 22;7(2):e31917. doi: 10.1371/journal.pone.0031917 (PMC3284524; doi:10.1371/journal.pone.0031917)

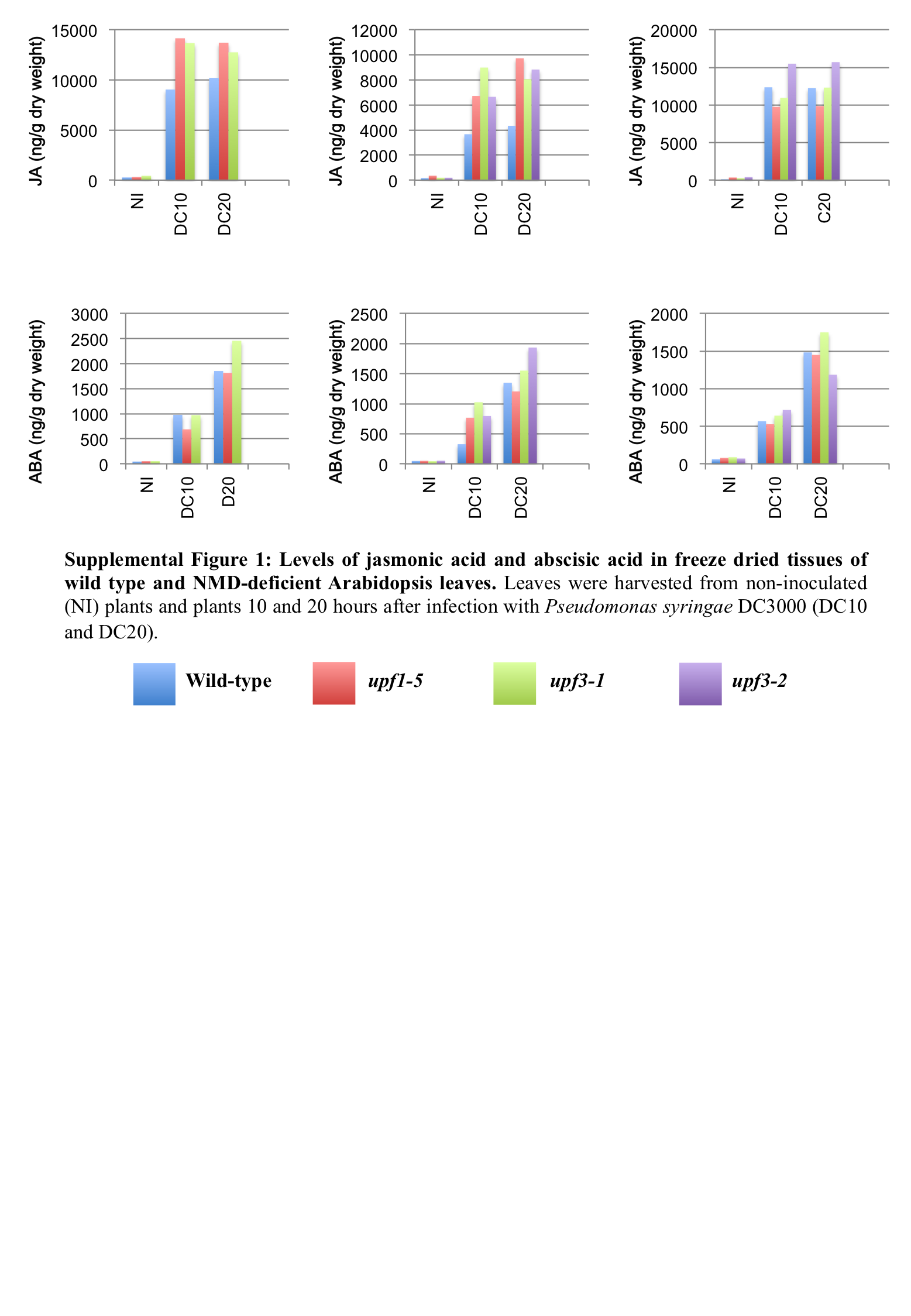

Supplement: Figure S1 — Levels of jasmonic acid and abscisic acid in freeze dried tissues of wild type and NMD-deficient Arabidopsis leaves. Leaves were harvested from non-inoculated (NI) plants and plants 10 and 20 hours after infection with Pseudomonas syringae DC3000 (DC10 and DC20). Wild-type plants are represented by blue bars, upf1-5 mutants by red bars, upf3-1 by green bars and upf3-2 plants by purple bars. (TIFF) [file pone.0031917.s001.tif]
